# Serum hepatitis B surface antigen levels predict insignificant fibrosis and non-cirrhosis in hepatitis B e antigen positive patients with normal or mildly elevated alanine transaminase levels

**DOI:** 10.18632/oncotarget.21210

**Published:** 2017-09-23

**Authors:** Qiang Li, Weixia Li, Chuan Lu, Yuxian Huang, Liang Chen

**Affiliations:** ^1^ Department of Hepatitis, Shanghai Public Health Clinical Center, Fudan University, Shanghai 201508, China; ^2^ Department of Infectious Disease, Huashan Hospital, Fudan University, Shanghai 200040, China

**Keywords:** chronic hepatitis B, liver fibrosis, cirrhosis, non-invasive marker, hepatitis B surface antigen

## Abstract

Background/Aims: We aimed to evaluate the diagnostic value of serum hepatitis B surface antigen (HBsAg) levels for liver fibrosis in hepatitis B e antigen-positive [HBeAg (+)] chronic hepatitis B (CHB) patients with alanine transaminase (ALT)≤twice upper limit of normal (ULN). Methods: 505 patients who underwent liver biopsies and HBsAg quantitative detections were included. Liver histology was scored using METAVIR scoring system. The area under the receiver-operator curve (AUROC) was used to determine the diagnostic accuracy. Results: Of 505 CHB patients, 333 have HBeAg (+), and 172 have HBeAg (-). HBsAg levels and METAVIR fibrosis scores showed strong correlation (r=-0.50, *p*<0.001) in HBeAg (+) patients, but no correlation in HBeAg (-) patients (r=0.09, *p*=0.239). HBeAg (+) patients with insignificant fibrosis (F0-1) exhibited higher HBsAg levels than those with significant fibrosis (F2-4) (4.60 *vs* 4.12 log10IU/ml, *p*<0.001). HBeAg (+) patients with non-cirrhosis (F0-3) exhibited higher HBsAg levels than those with cirrhosis (F4) (4.48 *vs* 3.95 log10IU/ml, *p*<0.001). In this study, the AUROC of HBsAg was 0.86 for diagnosing insignificant fibrosis, and 0.91 for diagnosing non-cirrhosis in HBeAg (+) CHB patients. Conclusions: Serum HBsAg level can identify insignificant fibrosis and non-cirrhosis in HBeAg (+) CHB patients with ALT≤2 ULN, and thus avoid liver biopsy in this population.

## INTRODUCTION

Approximately 240 million people are hepatitis B virus (HBV) surface antigen (HBsAg) carriers worldwide [1]. The number of HBV related deaths due to liver cirrhosis and/or hepatocellular carcinoma (HCC) increased between 1990 and 2013 by 33%, relating to over 686,000 cases in 2013 worldwide [1, 2]. Chronic HBV infection has a relatively low prevalence in Europe and the USA, but intermediate to high prevalence in the Asia-Pacific region. In China, the prevalence of HBsAg is 9.75% in 1992, and 7.18% in 2006 [3]. According to the international guidelines [1, 4, 5], CHB patients with significant fibrosis or cirrhosis should receive antiviral therapy, and HCC surveillance is mandatory for all patients with cirrhosis. Thus, the assessment of the severity of liver fibrosis is important to identify patients for treatment and HCC surveillance.

Liver biopsy is the gold standard for the assessment of liver fibrosis severity, but limited by expensive and invasive procedure, patient discomfort, and a risk of serious bleeding [6]. Recently, the development of algorithms based on serum markers and transient elastography for detecting liver fibrosis and cirrhosis decreases the use of liver biopsy [7]. However, in CHB patients, the currently available approaches are suboptimal partially because of poor specificity, and easy to be interfered by various factors [8]. Therefore, HBV-specific fibrosis markers are expected to be used as the non-invasive diagnostic tests for liver fibrosis and cirrhosis in CHB patients.

HBsAg is secreted from HBV-infected liver cells and circulates within the serum. HBsAg is specific to HBV infection, and generally considered as a diagnostic tool for HBV infection [9]. In recent years, the availability of commercial quantitative assays reinforced the value of HBsAg for the diagnosis and treatment of CHB patients. A French study showed that serum HBsAg level is associated with fibrosis severity in hepatitis B e antigen-positive [HBeAg (+)] CHB patients [8]. Xun et al found that serum HBsAg quantification was a useful marker for significant fibrosis in HBeAg (+) CHB patients, and 80% of liver biopsy could be avoided in the population [10]. Seto et al found that high HBsAg levels can predict insignificant fibrosis (F0-1) in HBeAg (+) patients [11], which was consistent with the results of A Chinese study [12].

Although several studies have evaluated the association between serum HBsAg levels and the fibrosis severity in HBeAg (+) CHB patients [8, 10–13], but limited by small sample sizes. Besides, previous studies were restricted to selected HBeAg (+) patients and did not include analysis of HBeAg (-) patients [10–13]. In China, only two small sample sizes studies evaluated the diagnostic value of HBsAg levels for liver fibrosis in CHB patients [12, 13]. This study aimed to investigate the diagnostic value of HBsAg levels for liver fibrosis in a large cohort of 505 CHB patients with normal or mildly elevated alanine transaminase (ALT) levels.

## RESULTS

### Study population

The baseline demographics of the study population were showed in Table 1. 505 CHB patients were included: 333 patients (65.9%) were HBeAg (+), and 172 patients (34.1%) were HBeAg (-). Most patients were male in HBeAg (+) group (57.7%) and HBeAg (-) group (61.6%). HBeAg (+) group has higher HBsAg (4.45 *vs* 3.25 log10IU/ml, *p*<0.001), HBV DNA (6.95 *vs* 3.80 log10 copies/ml, *p*<0.001), and ALT (39 *vs* 31 IU/L, *p*<0.001) levels than HBeAg (-) group.

**Table 1:** Patient characteristics

Patient group	All, (N=505)	HBeAg(+), (N=333)	HBeAg(-), (N=172)	*p* value
Age (years)	35 (28-42)	35 (28-42)	36 (30-42)	0.275
Male, n (%)	301 (59.6%)	192 (57.7%)	109 (61.6%)	0.162
HBsAg (log10 IU/ml)	4.14 (3.62-4.58)	4.45 (4.15-4.70)	3.25 (2.79-3.69)	<0.001
HBVDNA (log10 copies/ml)	5.84 (4.05-7.52)	6.95 (5.27-7.70)	3.80 (3.29-4.66)	<0.001
ALT (IU/L)	35 (26-53)	39 (27-54)	31 (22-47)	<0.001
Liver inflammation stage				
A0	93 (18.4%)	53 (15.9%)	40 (23.3%)	0.044
A1	239 (47.3%)	156 (46.8%)	83 (48.3%)	0.764
A2	122 (24.2%)	92 (27.6%)	30 (17.4%)	0.011
A3	51 (10.1%)	32 (9.6%)	19 (11.0%)	0.612
Liver fibrosis stage				
F0-1	360 (71.3%)	239 (71.8%)	121 (70.3%)	0.738
F2-4	145 (28.7%)	94 (28.2%)	51 (29.7%)	0.738
F3-4	74 (14.7%)	42 (12.6%)	32 (18.6%)	<0.001
F4	41 (8.1%)	20 (6.0%)	21 (12.2%)	<0.001

HBsAg, hepatitis B surface antigen; HBeAg, hepatitis B e antigen; ALT, alanine transaminase; METAVIR scoring system was used to determine the liver inflammation and fibrosis stages

HBeAg (+) group has lower prevalence rate of F3-4 (12.6% *vs* 18.6%, *p*<0.001) and F4 (6.0% *vs* 12.2%, *p*<0.001) compared with HBeAg (-) group. There were no significant differences in the prevalence rate of F0-1 (71.8% *vs* 70.3%, *p*=0.738) and F2-4 (28.2% *vs* 29.7%, *p*=0.738) between the two patient groups.

### Correlation between serum HBsAg and HBV DNA levels

**Figure 1:**
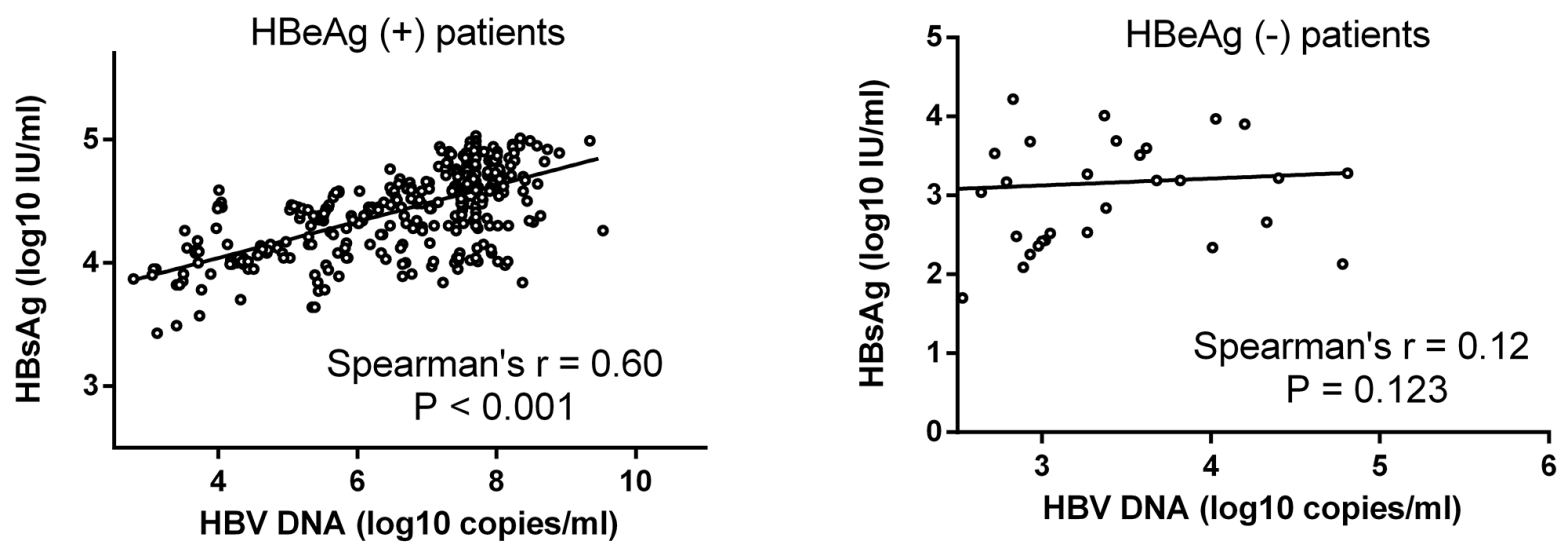
Correlation between serum HBsAg and HBV DNA levels Correlation was evaluated within the HBeAg (+) patient group and the HBeAg (-) patient group. There was a strong correlation between serum HBsAg and HBV DNA levels in HBeAg (+) patients (r=0.60, *p*<0.001) but no correlation in HBeAg (-) patients (r=0.12, *p*=0.123).

The relationship between serum HBsAg and HBV DNA levels was showed in Figure 1. Stratifying the patients according to HBeAg status revealed a strong correlation between HBsAg and HBV DNA levels in HBeAg (+) patients (r=0.60, *p*<0.001) but no correlation in HBeAg (-) patients (r=0.12, p=0.123).

### Correlation between serum HBsAg levels and METAVIR fibrosis scores

**Figure 2:**
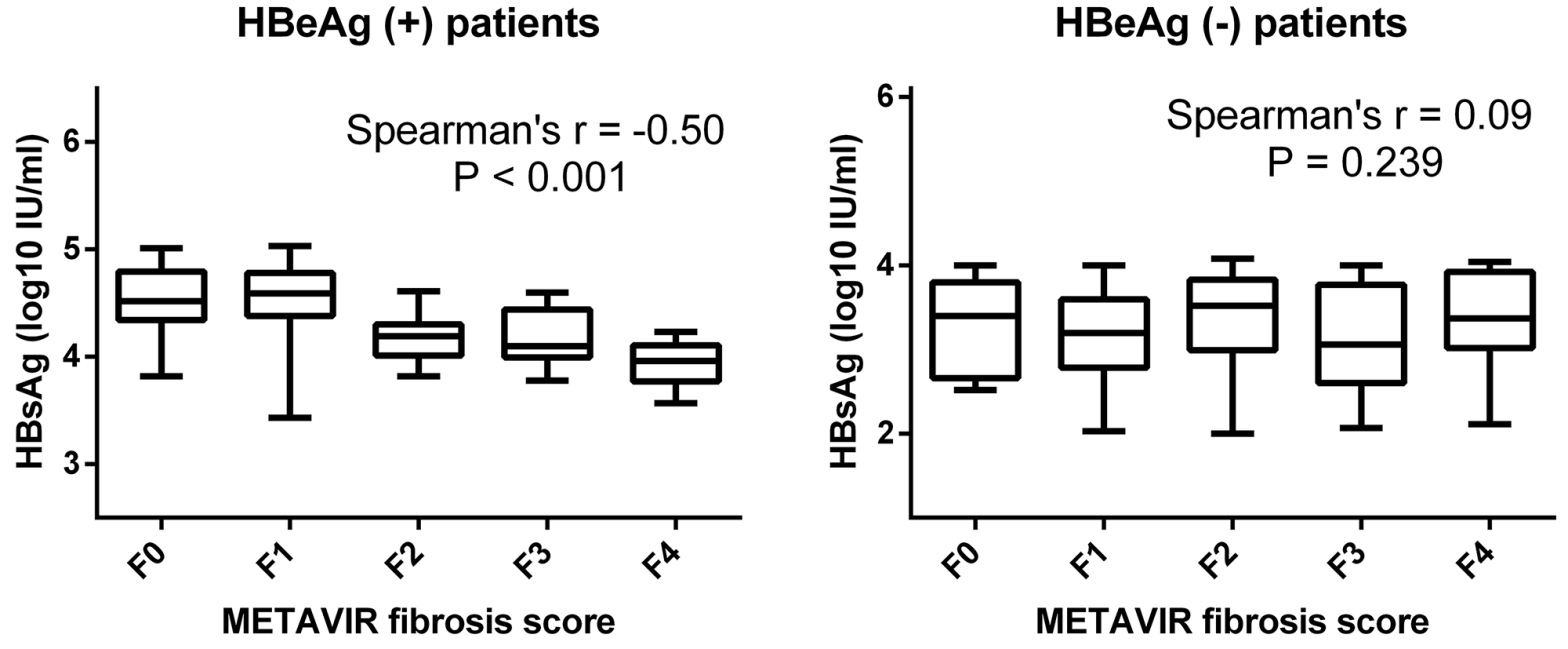
Correlation between serum HBsAg levels and METAVIR fibrosis scores Serum HBsAg levels in HBeAg (+) and HBeAg (-) patients were stratified according to METAVIR fibrosis scores. There was a strong correlation between serum HBsAg levels and METAVIR fibrosis scores in HBeAg (+) patients (r=-0.50, *p*<0.001) but no correlation in HBeAg (-) patients (r=0.09, *p*=0.239).

The relationship between serum HBsAg levels and METAVIR fibrosis scores was showed in Figure 2. Stratifying the patients according to HBeAg status revealed a strong correlation between serum HBsAg levels and METAVIR fibrosis scores in HBeAg (+) patients (r=-0.50, *p*<0.001) but a lack of correlation in HBeAg (-) patients (r=0.09, *p*=0.239). Increasing severity of liver fibrosis in HBeAg (+) patients was associated with a decreasing serum level of HBsAg levels.

### Serum HBsAg levels can identify HBeAg (+) patients with F0-1, F0-2, and F0-3

**Figure 3:**
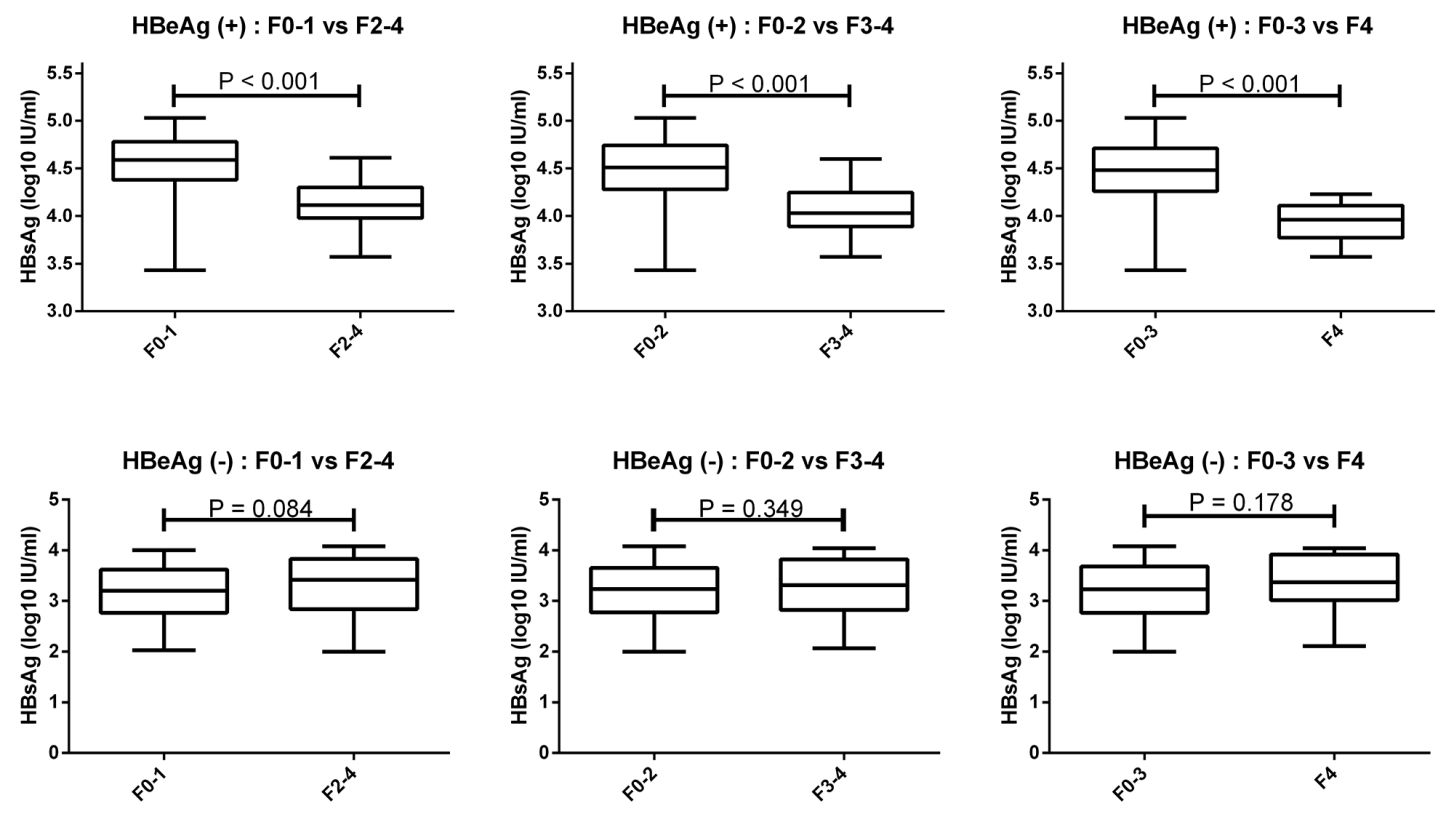
Serum HBsAg levels can identify HBeAg (+) patients with F0-1, F0-2, and F0-3 HBeAg (+) patients with F0-1 exhibited higher HBsAg levels compared with F2-4. HBeAg (+) patients with F0-2 exhibited higher HBsAg levels compared with F3-4. HBeAg (+) patients with F0-3 exhibited higher HBsAg levels than F4. There was no significant difference in serum HBsAg levels of HBeAg (-) patients with F0-1 compared with F2-4, as well as of F0-2 compared with F3-4, and F0-3 compared with F4.

The serum HBsAg levels according to HBeAg status and fibrosis stage were showed in Table 2 and Figure 3. HBeAg (+) patients with F0-1 exhibited a significantly higher HBsAg levels compared with patients with F2-4 (4.60 *vs* 4.12 log10IU/ml, *p*<0.001). HBeAg (+) patients with F0-2 exhibited a significantly higher HBsAg levels compared with patients with F3-4 (4.51 *vs* 4.03 log10IU/ml, *p*<0.001). HBeAg (+) patients with F0-3 exhibited a significantly higher HBsAg levels compared with patients with F4 (4.48 *vs* 3.95 log10 IU/ml, *p*<0.001).

**Table 2:** The serum levels of HBsAg (log10IU/ml) according to HBeAg status and METAVIR fibrosis stage.

	F0-1	F2-4	P value	F0-2	F3-4	P value	F0-3	F4	P value
HBeAg (+)	4.60 (4.38-4.78)	4.12 (3.98-4.30)	<0.001	4.51 (4.28-4.74)	4.03 (3.89-4.25)	<0.001	4.48 (4.26-4.71)	3.95 (3.74-4.10)	<0.001
HBeAg (-)	3.20 (2.77-3.62)	3.42 (2.84-3.83)	0.084	3.32 (2.82-3.82)	3.24 (2.78-3.65)	0.349	3.23 (2.77-3.68)	3.37 (3.02-3.92)	0.178

HBsAg, hepatitis B surface antigen; HBeAg, hepatitis B e antigen;

### Predictive value of serum HBsAg levels for F0-1, F0-2, and F0-3 in HBeAg (+) CHB patients

**Figure 4:**
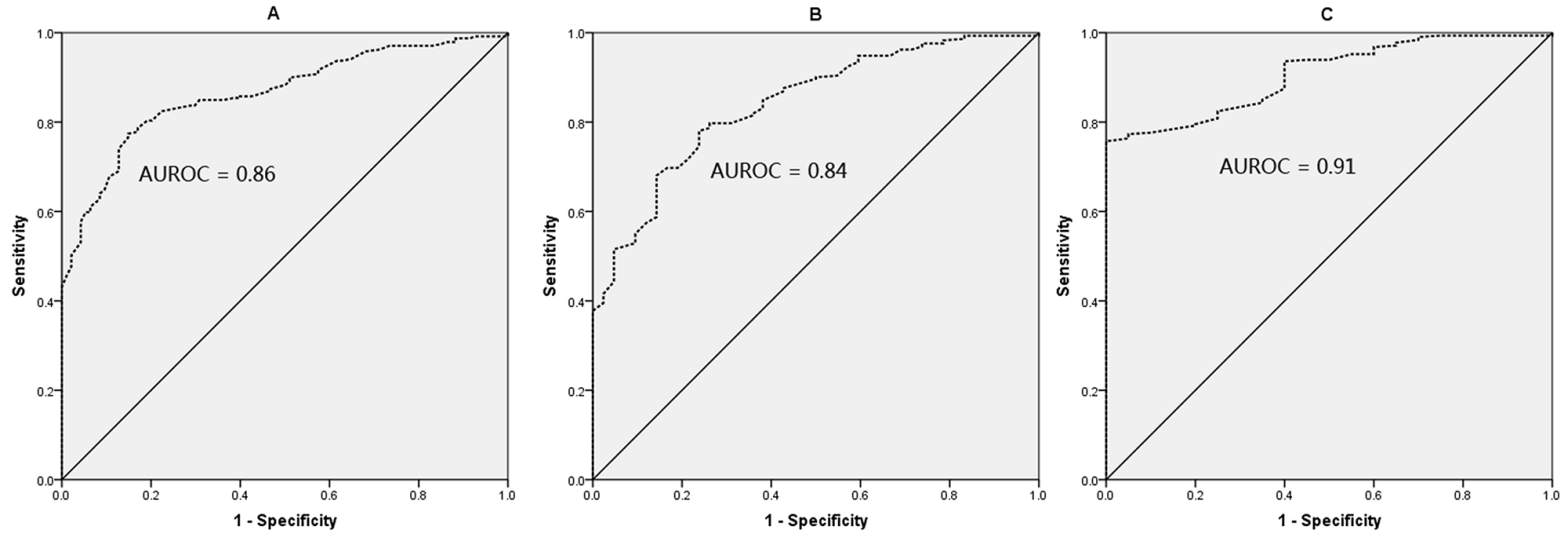
ROC curves of HBsAg for F0-1 **(A)**, F0-2 **(B)**, and F0-3 **(C)** in HBeAg **(+)** patients. The AUROC was 0.86, 0.84, and 0.91, respectively, to predict F0-1, F0-2, and F0-3.

The ROC curves of HBsAg levels for predicting F0-1, F0-2, and F0-3 in HBeAg (+) CHB patients were showed in Figure 4. The AUROC was 0.86, 0.84, and 0.91, respectively, to predict F0-1, F0-2, and F0-3. According to maximizing Youden index, the optimal cut-off of HBsAg was 4.36 log10IU/ml for diagnosing F0-1 (the sensitivity, specificity, PPV, and NPV was 77%, 85%, 93%, and 60%, respectively), and 4.23 log10IU/ml for diagnosing F0-3 (the sensitivity, specificity, PPV, and NPV was 76%, 95%, 99%, and 21%, respectively), in HBeAg (+) CHB patients (Table 3).

**Table 3:** Cut-offs of HBsAg for predicting F0-1 and F0-3 in HBeAg positive patients

Classification	Cut-offs (log10IU/ml)	Youden index	Se, %	Sp,%	PPV, %	NPV,%
F0-1	≥4.36	0.62	77	85	93	60
F0-3	≥4.23	0.76	76	95	99	21

HBsAg, hepatitis B surface antigen; HBeAg, hepatitis B e antigen; Se, sensitivity; Sp, specificity; PPV, positive predictive value; NPV, negative predictive value; Cut-offs were established by maximizing Youden index.

## DISCUSSION

We observed a positive correlation between serum HBsAg and HBV DNA levels in HBeAg (+) CHB patients with ALT≤2 ULN, as well as a negative correlation between HBsAg levels and the severity of fibrosis. However, the correlations were lacking in HBeAg (-) patients. The results were consistent with previous studies [14–16]. A European study showed that serum HBsAg levels had a strong correlation with HBV-DNA levels (R=0.79, *p*<0.01), and HBeAg (+) patients had higher serum HBsAg levels than HBeAg (-) patients [14]. An Asia study also showed that serum HBsAg levels correlated with HBV DNA levels in immune-tolerant phase [15]. A study from Australia found that serum HBsAg levels were positively correlated with HBV DNA levels (r=0.69, *p*<0.01) in HBeAg (+) patients [16].

According to CHB guidelines, the indications for antiviral therapy are generally the same for both HBeAg (+) and HBeAg (-) patients, which is based mainly on the combination of three criteria: serum HBV DNA levels, serum ALT levels, and severity of liver histology including inflammation and fibrosis [1, 4, 5]. Patients with HBV DNA > 20,000 IU/ml and ALT > 2 ULN can start treatment even without an evaluation of severity of liver histology by liver biopsy. For these patients, liver biopsy does not change the decision for treatment [1]. In patients who have ALT≤2 ULN, treatment may be initiated when patients have significant liver inflammation and/or significant liver fibrosis. In patients who cannot or are reluctant to undergo liver biopsy, non-invasive tests of fibrosis may be used for decisions on treatment indications [1]. Generally speaking, patients with ALT≤2 ULN have more needs for liver fibrosis assessment than patients with ALT > 2 ULN. Therefore, we evaluated the diagnostic value of HBsAg for liver fibrosis in CHB patients with ALT≤2 ULN.

We found a significantly higher serum HBsAg levels in patients with F0-1 compared with those with F2-4 in HBeAg (+) patients. The mechanism for why the severity of liver fibrosis is associated with lower serum HBsAg levels in HBeAg (+) patients is unclear. A hypothesis might be reasonable, but need to be verified by experiments [8]: Martinot-Peignoux et al speculated that the decline in serum HBsAg levels with increasing severity of fibrosis may be due to the retention of HBsAg within cells rather than secretion, or a diminishing ability of the host to support viral replication with increasing severity of fibrosis. Alternatively, perhaps an increasingly humoral immune response with increasing severity of fibrosis complicates the serum HBsAg and causes it to become undetectable to the commercially-available assays. In addition, the dynamic evolution of the nature history of chronic HBV infection might be another reason for lower HBsAg along with development of fibrosis in HBeAg (+) patients. Chronic HBV infection is a dynamic process reflecting the dynamic relationship between viral replication and the host immune response. There is an increased likelihood of significant inflammation or fibrosis with transitioning from immune-tolerant to the immune-active phase where HBV virus is no doubt being cleared (if incompletely).

In this study, the optimal cut-offs of HBsAg were 4.36 and 4.23 log10IU/ml, respectively, for diagnosing F0-1, and F0-3, in HBeAg (+) CHB patients with ALT≤2 ULN. The study published by Seto et al found that the optimal cut-off of HBsAg was 4.4 log10IU/ml for diagnosing F0-1 in HBeAg (+) CHB patients with ALT≤2 ULN [11], which is consistent with our results. However, this study has several differences from the study by Seto et al [11]. First, Seto et al included only HBeAg (+) patients and did not report the association between HBsAg levels and fibrosis severity in HBeAg (-) patients. Second, Seto et al only included 140 patients, and a relatively small sample size could have resulted in statistical bias, whereas we included 505 patients. Third, Seto et al only evaluated the diagnostic value of HBsAg levels for insignificant fibrosis, whereas we evaluated the diagnostic value of HBsAg levels for non-cirrhosis and insignificant fibrosis.

In this study, using HBsAg≥4.36 log10IU/ml, 188/202 (93%) patients with F0-1 were correctly identified; using HBsAg≥4.23 log10IU/ml, 238/240 (99%) patients with F0-3 were correctly identified. These patients who were correctly identified therefore will avoid unnecessary liver biopsy. However, the NPV of HBsAg is 60% for F0-1, and 21% for F0-3. In this study, among 131 patients with HBsAg < 4.36 log10IU/ml, 53 (40%) patients have F0-1, and will be misdiagnosed as patients with F2-4 and might lead to unnecessary antiviral therapy. Among 93 patients with HBsAg < 4.23 log10IU/ml, 73 (79%) patients have F0-3, and will be misdiagnosed as patients with F4 and might lead to unnecessary therapy and HCC surveillance.

This study has several limitations. Firstly, HBV genotypes were unavailable in this study. One study showed that there may be a link between HBV genotype and the severity of fibrosis in HBeAg (+) patients [8]. It will be of considerable interest to further analyze fibrosis severity in HBeAg (+) patients according to HBV genotype, and see whether a single cut-off is applicable to all HBV genotypes. Based on epidemiological evidence, genotypes B and C are common in China [17]. Thus, the cut-offs we have described will be applicable to HBV genotypes B or C at least. Secondly, information on transient elastography (or FibroScan) was unavailable in this study, and thus HBsAg levels had not been compared with FibroScan. The diagnostic value of combining HBsAg levels and FibroScan is not clear yet, and needs to be further investigated.

In conclusion, in a large cohort, we verified an association between serum HBsAg levels and the severity of liver fibrosis in HBeAg (+) CHB patients with ALT≤2 ULN. Furthermore, we describe a serum HBsAg cut-off for identifying insignificant fibrosis and non-cirrhosis in this population, who might avoid unnecessary liver biopsy. It is necessary to continue to monitor patients closely if the decision is not to treat.

## MATERIALS AND METHODS

### Patients

Five hundred and five consecutive CHB patients who underwent liver biopsies and HBsAg quantitative detections at Shanghai Public Health Clinical Center, Shanghai, China, between January 2010 and January 2017 were included. CHB was defined as the persistent presence of HBsAg for more than six months [1]. Inclusion criteria were: HBsAg-positive for more than six months, known HBeAg status, underwent liver biopses, known HBsAg quantitative value, ALT≤2 ULN (the ULN is 40 IU/L). Exclusion criteria were: a history of antiviral therapy, hepatitis C virus, hepatitis D virus, or HIV co-infection, alcohol consumption over 20g/day for more than 5 years, accompanied by nonalcoholic fatty liver disease or autoimmune liver disease.

All patients signed the informed consent before liver biopsy, and all clinical procedures were in accordance with the Helsinki declaration in 1983. The study protocol was permitted by the ethics committee of Shanghai Public Health Clinical Center.

### Laboratory tests

Serum HBsAg levels were quantified using the commercially available HBsAg QT assays (Abbott, Wiesbaden, Germany). The detection value ranges from 0.05 to 250 IU/mL, and serial 1:500 dilutions were performed if the HBsAg titers were > 250 IU/mL. Serum HBV DNA was quantified by real-time PCR (Applied Biosystems, Foster City, USA) with the detection limit 500 copies/ml. Serum ALT was measured by biochemistry analyzer (Hitachi, Tokyo, Japan).

### Liver histological assessment

Liver biopsy was performed under local anesthesia. Liver samples were fixed in 10% formalin, paraffin-embedded, and stained with hematoxylin and eosin. A minimum of 15mm of liver tissue with at least six portal tracts were required for histological scoring [18]. The METAVIR scoring system was used to determine the severity of liver inflammation and fibrosis [19]. Liver inflammation was divided into four stages: A0, none inflammation; A1, mild inflammation (focal, few portal areas); A2, moderate inflammation (most portal areas, and even extended to beyond the portal areas); and A3, severe inflammation (significant confluent necrosis and bridging necrosis). Fibrosis stage was determined: F0, no fibrosis; F1, portal fibrosis without septa; F2, portal fibrosis with rare septa; F3, numerous septa without cirrhosis; and F4, cirrhosis. We defined insignificant fibrosis as F0-1, significant fibrosis as F2-4, non-cirrhosis as F0-3, and cirrhosis as F4.

### Data analysis

Kolmogorov-Smirnov test was used to verify the normal assumption of quantitative data. All quantitative data in this study was non-normal distribution data, and was presented as median (interquartile range). All qualitative data was presented as number (percentage). Statistical differences between two groups were tested using Chi-square test for qualitative data, and Mann Whitney test for non-normal distribution quantitative data. Spearman’s correlation coefficient was used for correlation analysis. Area under receiver-operator curve (AUROC) was used to calculate the diagnostic accuracy, and sensitivity, specificity, positive predictive value (PPV), and negative predictive value (NPV) for relevant cut-off was displayed [20]. The optimal cut-off was calculated by maximizing Youden index (sensitivity+specificity-1). All tests were two-sided and used a significance level of 0.05. All statistical analyses were carried out using the SPSS statistical software version 15.0 (SPSS Inc. Chicago, Illinois, USA) and MedCalc Statistical Software version 16.1 (MedCalc Software bvba, Ostend, Belgium).
